# A Green Innovative Approach for Solubility Enhancement of Poorly Water-Soluble Drugs Using Choline Chloride–Polyol Eutectic Solvents

**DOI:** 10.3390/ijms27073110

**Published:** 2026-03-29

**Authors:** Liga Petersone, Rihards Mahinovs, Zoltán Márk Horváth, Valentyn Mohylyuk

**Affiliations:** Leading Research Group, Faculty of Pharmacy, Rīga Stradiņš University, LV-1007 Riga, Latvia

**Keywords:** eutectic solvents, green solution, choline chloride, polyols, sustainable process, poorly water-soluble drugs, solubility

## Abstract

Eutectic solvents have become a viable choice to create innovative pharmaceutical technologies within the framework of the green chemistry approach. Despite the growing applicative interest, a general gap remains in the pharmaceutical sector regarding thorough and systematic research of their properties and useful applications. In this work, eutectic solvents have been prepared from choline chloride and polyols (sorbitol, xylitol, mannitol, and isomalt) at different molar ratios (1:1, 2:3, and 3:2), characterised, and used for the solubility enhancement of poorly water-soluble drugs (ibuprofen and naproxen) as well as the potential drug candidate apigenin. The interactions between the eutectic solvent components were investigated by DSC, FTIR, and refractive index methods. In all eutectic solvents, the water content detected by Karl Fischer titration and loss on drying was less than 3%. Solubility studies, carried out using the shake-flask method, showed significant solubility enhancement of the following: ibuprofen: ~152-fold increase, naproxen: ~144-fold increase, and apigenin: ~188-fold increase. These findings highlighted the great potential of eutectic solvents as solubility enhancers in the development of novel and more effective drug delivery systems.

## 1. Introduction

The pharmaceutical sector is facing increasing criticism for its environmental impact, despite its critical role in improving global health. Sustainability is an important challenge, as it involves ensuring access to essential pharmaceuticals while reducing environmental impact [1]. Overall, the pharmaceutical industry is moving in the right direction regarding green chemistry, and eco-friendly chemical products have gained popularity in pharmacy, including the replacement of hazardous chemicals with safer alternatives wherever feasible [2].

The role of eutectic solvents (ESs) in pharmaceutical formulation and drug delivery is rapidly expanding, particularly in the development of novel green approaches to obtain modern poorly water-soluble drug-based pharmaceuticals. It should be noted that not all solubility-enhancement methods or solvents are suitable and desirable for pharmaceutical applications [3]. ESs represent a relatively new class of green solvents formed by combining two or more organic compounds. They comprise hydrogen bond acceptors (HBAs) and hydrogen bond donors (HBDs), where the physicochemical properties can be tailored by selecting combinations of distinct HBAs and HBDs [4]. ESs have melting temperature values that are significantly lower than those of their individual pure organic components. At room temperature, they exist as liquids composed of a mixture of HBAs and HBDs in a specific molar ratio [5]. By combining two or more components in a specific ratio, eutectic mixtures can provide improved solubility, stability, and dissolution rates, leading to enhanced drug performance and patient outcomes [6]. Suppositories, tablets, capsules, and creams are just a few examples of potential dosage forms that eutectic formulations can be manufactured into [6].

Eutectic-based formulations are a promising strategy for improving the solubility and bioavailability of poorly soluble drugs in solid dosage forms such as tablets. The dissolution rate and oral absorption of solid dosage forms can be enhanced by the addition of eutectic mixtures to the formulations [7]. The potential of ESs to generate low-viscosity liquid phases makes them a perfect candidate for cutaneous and mucosal applications, since it also makes it easier for drugs to pass through biological membranes [8]. Furthermore, eutectic solutions frequently include pharmaceutically acceptable and biocompatible excipients, enabling patient-friendly and economical formulation techniques [9].

Polyols are an excellent green chemistry solution for pharmacy; regarding their functionality, they could be utilised not only as excipients and active ingredients, but also as innovative components of drug delivery systems [10,11]. Pharmaceutically applicable polyols are derived from renewable agricultural raw materials, which provide them with a “natural” and “green” status [12]. Despite the many applications of polyols, ongoing research continues to focus on fully understanding their functionality and exploring new ways to utilise their unique characteristics to improve pharmaceutical products [13].

Choline chloride (ChCl) is widely used in the development of ESs due to its unique structure (bifunctional compound, quaternary ammonium functional group, and hydroxyl functional group). ChCl is generally accessible, easy to handle, relatively cheap, nontoxic, and moisture-stable, which fulfils several principles of green chemistry [14,15]. The real advantage of ChCl-based ES is in the phase separation, which can be isolated easily by decantation [16,17]. In eutectic mixtures, ChCl functions as an HBA with a variety of HBDs, such as urea, alcohols, hydroxy acids, amino acids, and sugars/polyols [18,19].

Many newly developed and currently available drugs are categorised as class II compounds according to the Biopharmaceutical Classification System (BCS) due to their poor water solubility [20,21]. Therefore, in this study, the model compounds of poorly water-soluble drugs were selected from BCS class II, such as ibuprofen, apigenin, and naproxen.

This work focuses on the use of safer and more environmentally friendly solvents in accordance with the 12 principles of green chemistry. In the upcoming years, eutectic solvents may contribute to reducing the use and waste from organic solvents. Compared to standard solvents, they are renewable, biodegradable, and non-toxic, which makes them an appealing alternative for a pharmaceutical application [22,23].

Eutectic solvents are becoming increasingly important in green chemistry due to their unique properties, such as easy synthesis, low cost, environmental friendliness, low volatility, high dissolution power, high biodegradability, and feasibility of structural design [23,24].

Eutectic solvents are promising biocompatible alternatives for pharmaceutical applications, especially when it comes to improving solubility and stability, and acting as possible drug delivery vehicles for active pharmaceutical components [25]. ESs are investigated within the pharmaceutical industry to tackle one of its biggest issues, poor solubility in water [9,26].

This research aims to improve the solubility of poorly water-soluble drugs (specifically, ibuprofen, naproxen, and apigenin) by using innovative formulations of choline chloride-polyol eutectic solvents as green solvents.

## 2. Results and Discussion

Green chemistry concepts, which prioritise components that are cost-effective, pharmaceutically acceptable, and environmentally friendly, were crucial in the component selection process. The selection of components for eutectic solvent formulations was guided by the aim of enhancing the solubility of the model drugs (ibuprofen, naproxen, and apigenin).

Choline chloride and various polyols (sorbitol, xylitol, mannitol, and isomalt) were selected as the most suitable compounds for the preparation of ESs, as they met pharmaceutical requirements and aligned with the green chemistry concept. Sucrose was taken as a carbohydrate reference compound for comparison with sugar alcohols (polyols).

All selected model compounds belong to the BCS class II and exhibit poor water solubility, making their preparation in liquid solutions challenging. As a result, the market offers suspensions but not clear liquid solutions. Furthermore, drug suspensions are often prepared with excipients that may be inappropriate for certain populations, for example, sugar for patients with diabetes or fructose intolerance, and alcohol for paediatric patients.

Ibuprofen and naproxen, well-known synthetic drugs, were selected due to their poor aqueous solubility within the physiological pH range. Apigenin was chosen as a poorly soluble natural drug candidate to investigate the potential of ESs for solubility enhancement of “natural drugs”. This study aims to expand the application of ESs not only to pharmaceutical drugs but also to health-promoting agents, such as food supplements.

**Figure 1:**
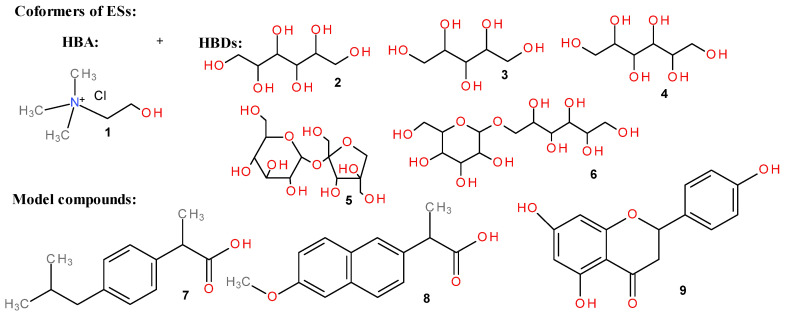
Structural formulas of choline chloride (**1**), sorbitol (**2**), xylitol (**3**), mannitol (**4**), sucrose (**5**), isomalt (**6**), ibuprofen (**7**), naproxen (**8**), and apigenin (**9**).

The structural formulas of the ES components and model compounds used in this research are shown in Figure 1.

Our research presents quite new ES systems, broadening the range of pharmaceutically acceptable ES formulations, showing new opportunities to improve drug solubility. ES formulations obtained in this research are made of ingredients with proven safety profiles and known applications in pharmaceutical products.

### 2.1. ES Solid–Liquid Equilibria (Pre-Screening)

The preparation of ESs requires an understanding of solid–liquid equilibria in order to select the proper HBD:HBA molar ratio. The fusion characteristics, rather than the interactions between the HBD:HBA, dictate the eutectic composition [27]. Two melting curves intersect to produce the eutectic composition [28]; this cross point has the lowest melting point on the phase diagram [29]. In this research, the two pure compounds (ChCl and various polyols) were mixed with a mortar and pestle with no time left for interaction. The precise melting point could not be detected by the melting point apparatus. Although the constituents of the DES were mixed, they were not heated for long enough to form strong interactions. However, the behaviour of the mixtures indicated the temperature at which the melting point was lowest, corresponding to the strongest interaction.

**Figure 2:**
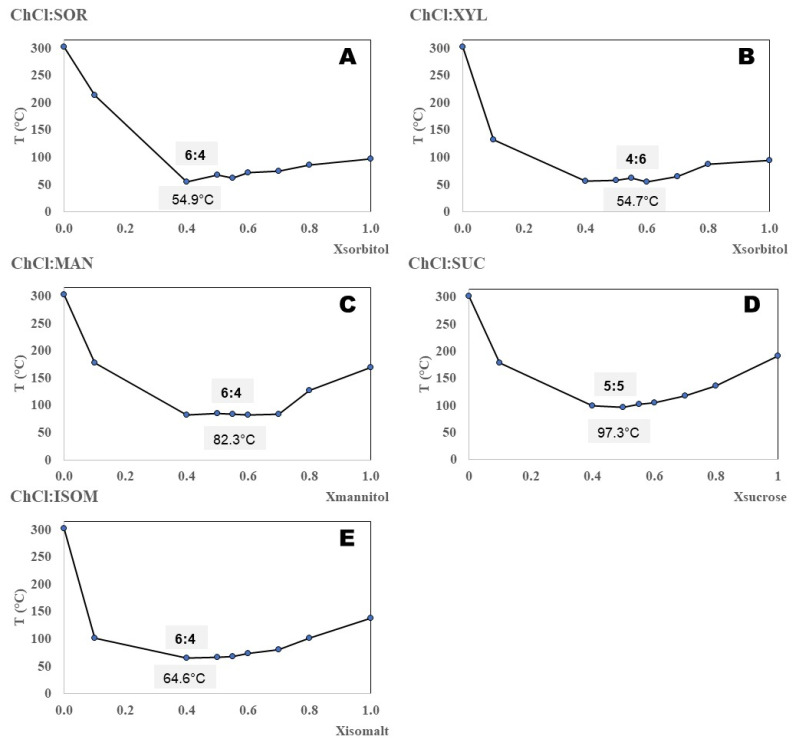
Solid–liquid phase diagrams for DES composed of choline chloride (ChCl) and different polyols: sorbitol (SOR) (**A**), xylitol (XYL) (**B**), mannitol (MAN) (**C**), sucrose (SUC) (**D**), and isomalt (ISOM) (**E**). The results are based on measurements obtained using a melting point apparatus.

Solid–liquid phase diagrams were constructed using a melting point apparatus as a preliminary screening technique to identify the minimum melting temperature of physical mixtures of eutectic-forming compounds. The five solid–liquid phase diagrams constructed in this research are shown in Figure 2. (Raw data in Table S1 of Supporting Information). All ES systems present a solid–liquid phase diagram characterised by a single eutectic point. The phase diagrams indicate that the formed mixtures are eutectic solvents, with the eutectic point above room temperature. However, based on the observed melting point depressions, some systems exhibit larger-than-expected decreases relative to idealised mixtures, suggesting that their classification as deep eutectic solvents (DESs) warrants further consideration.

To the best of our knowledge, literature data are available for ChCl + sorbitol, ChCl + xylitol, ChCl + mannitol, and ChCl + sucrose systems, but not for ChCl + isomalt systems.

All systems obtained presented an eutectic point close to equimolar composition. The data measured in this work are compared with values reported in the literature (Table 1).

### 2.2. Eutectic Solvent Preparation and Characterisation

To determine the optimal molar ratio corresponding to the lowest melting point, additional mixtures with molar ratios slightly below and above the detected equilibrium (by the melting point apparatus) were prepared. The ESs were prepared by adding an appropriate amount of HBD and HBA into a flask, which was later heated and stirred until the formation of a liquid. Since there were no by-products, the ES preparation generated zero waste and was inexpensive, showing good potential for large-scale use.

Most of the polyol-based ES remained semi-solid or liquid with high viscosity at room temperature (Table S2 of Supporting Information).

#### 2.2.1. Differential Scanning Calorimetry (DSC)

The main method for characterising ES was differential scanning calorimetry (DSC), which was used mainly to determine the eutectic temperature, melting point, and glass transition behaviour of ESs. DSC verified the creation of a new phase with a lower melting point than its constituent parts by the combination of components (such as hydrogen bond donors like polyols and acceptors like choline chloride) [34].

**Figure 3:**
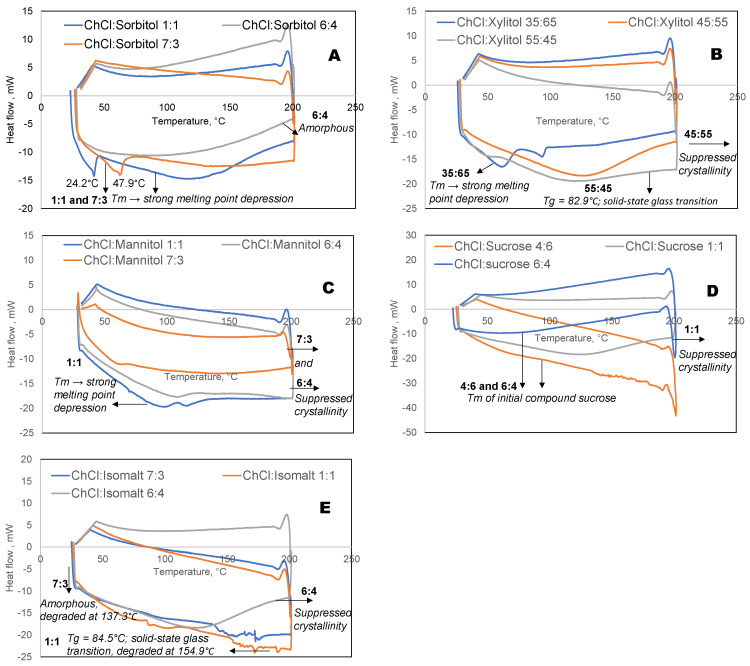
DSC curves of eutectic solvents prepared from choline chloride and various polyols: sorbitol (**A**), xylitol (**B**), mannitol (**C**), sucrose (**D**), and isomalt (**E**). The curves show the melting transitions of the eutectic mixtures, with the observed depressions in melting points indicating the formation of hydrogen-bonded eutectic systems.

The melting points of the prepared eutectic solvents were determined using differential scanning calorimetry (DSC) over a temperature range of 25–200 °C at a heating rate of 10 °C/min under a nitrogen atmosphere. The thermograms obtained from the DSC analysis of eutectic solvents (Figure 3) did not present the characteristic peaks of the individual pure components (Figure S1). The eutectic mixtures around the eutectic point showed a strong melting point depression (Figure 3). It should be noted that amorphous eutectic systems generally exhibit faster degradation than semi-crystalline counterparts due to increased molecular mobility and higher thermodynamic energy [35,36].

It should be noted that the true melting point of choline chloride cannot be determined under conventional experimental conditions due to thermal decomposition. Therefore, the commonly reported temperature of 301–302 °C corresponds to its decomposition rather than melting. The true melting point determined by ultrafast calorimetry in non-equilibrium conditions reported by Van den Bruinhorst et al. [37] is appreciably higher at 413 °C.

The temperature of chloride described in this study is its decomposition temperature rather than its actual melting point. When handling and applying choline chloride in eutectic solvents, this value is utilised to represent realistic experimental conditions.

Considering that ES are formed via hydrogen bonding networks between donor and acceptor, Fourier Transform Infrared Spectroscopy (FTIR) was applied to experimentally verify the bonding sites and hydrogen bonding.

#### 2.2.2. Intermolecular Structure Characterised by Infrared Spectroscopy (FTIR)

**Figure 4:**
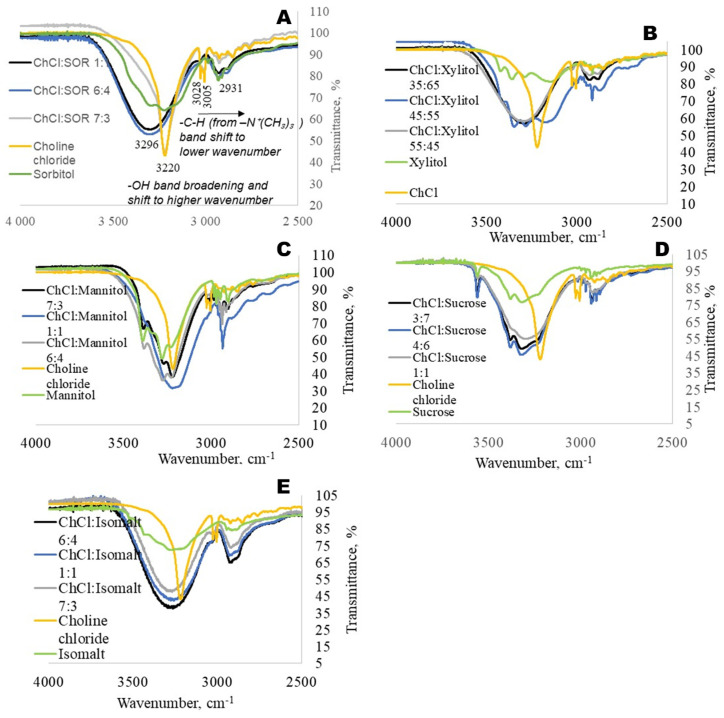
FTIR spectra (4000–2500 cm^−1^) of the HBA and HBD groups in eutectic solvents prepared from choline chloride and various polyols: sorbitol (**A**), xylitol (**B**), mannitol (**C**), sucrose (**D**), and isomalt (**E**).

In these studies, the intermolecular interactions of the compounds in the eutectic solvents were characterised by Fourier-transform infrared (FTIR) spectroscopy over the range 400–4000 cm^−1^, with a spectral resolution of 4 cm^−1^. In the choline chloride molecule, the chloride ion is the primary HBA [38]. The Cl^−^ acts as a bridge between the choline part (Ch+) and the corresponding donors, and Ch+ exhibits strong intermolecular interactions with the donor molecules’ acceptor groups, such as C=O and O–H [39]. It is already reported that the primary hydrogen bonding between choline chloride and polyols, like ethylene glycol, is formed between the polyol -OH groups and the Cl- anion of choline chloride [40]. The eutectic solvents formed from choline chloride and polyols (at a specific molecular ratio) showed a large band at 3296 cm^−1^, which could be assigned to O-H groups of initial compounds. The hydroxyl group peak has been shifted to a higher wavenumber compared with pure polyol and choline chloride, and no peak of -OH from choline chloride was observed (Figure 4A). The C-H group from –N^+^(CH_3_)_3_ has been shifted to a lower wavenumber and has slight broadening. In eutectic solvents, the C–H stretching frequencies of methyl groups on quaternary ammonium nitrogen tend to shift to lower wavenumbers. Increased intermolecular interactions, such as hydrogen bonding between the ammonium cation and the anion (as in choline chloride-based systems), weaken the C–H bonds and cause this shift in the FTIR spectra [41].

The observed spectral shifts in the FTIR spectra could be explained by several factors related to the molecular environment and interactions within the samples. The observed FTIR shifts in the eutectic solvents are primarily due to hydrogen-bonding interactions between choline chloride and the polyol component. Specifically, the O–H stretching vibrations of the polyol and the N–H or O–H groups of choline chloride are perturbed, resulting in a shift to lower wavenumbers, which reflects the formation of strong hydrogen-bonding networks in the eutectic mixture.

When compared to smaller polyols, sucrose’s FTIR spectra display larger and slightly shifted O–H bands due to stronger hydrogen-bonding interactions with choline chloride. These spectral changes collectively confirm that hydrogen bonding is the primary intermolecular interaction stabilising these eutectic systems.

The FTIR spectra indicate that a higher number of –OH groups results in broader and stronger hydrogen-bonding bands. The intensity and broadening of these bands decrease in the following order: sucrose, isomalt, mannitol, sorbitol, and xylitol.

#### 2.2.3. Loss on Drying, Water Content by Karl Fischer (KF) and pH Values of Eutectic Solvents

Loss on drying (LOD) and Karl Fischer titration are commonly used to determine the moisture content in pharmaceuticals [42]. Polyols and choline chloride are hygroscopic and readily absorb moisture from the air; therefore, determining their water content is essential for understanding ingredient fusion and explaining their behaviour.

Polyols can contain both absorbed (bound) water trapped within their structure and bulk (free) water, which can lead to a rapid increase in overall water content [43]. This water may help DESs maintain a structured hydrogen-bonded network, which would result in an environment that is beneficial for the solubilisation of hydrophobic drugs.

The moisture content determined through LOD was systematically higher for all ESs compared to the detected water content by Karl Fischer titration (Table 2). In addition to providing an estimate of water content, the LoD measurements can also serve as an indicator of the thermal stability of the prepared eutectic solvents, as higher mass loss at elevated temperatures may reflect decomposition or structural changes. Small mass changes of up to approximately 5% (Table 2) in the LoD measurements indicate that the eutectic solvent system remains thermally stable up to 230 °C.

It is known that the pH of DES-like mixtures can influence their stability, solvation properties, and reactivity [44]. Thus, the pH of mixtures was measured and systematised in Table 2. The results showed that the pH of the prepared ESs was neutral. This circumstance indicates a low risk of reactivity and uncompromised stability.

#### 2.2.4. Refractive Index

Refractive index is one of the key physical properties of deep eutectic solvents [45]. Depending on the component and the intermolecular interaction, raising the mole ratio may either raise or lower the refractive index of ES. The original and added components may have an impact on the refractive index; nonetheless, the presence of water lowers the refractive index of all ESs examined. When the temperature rises, the refractive index of ESs falls linearly [45].

**Figure 5:**
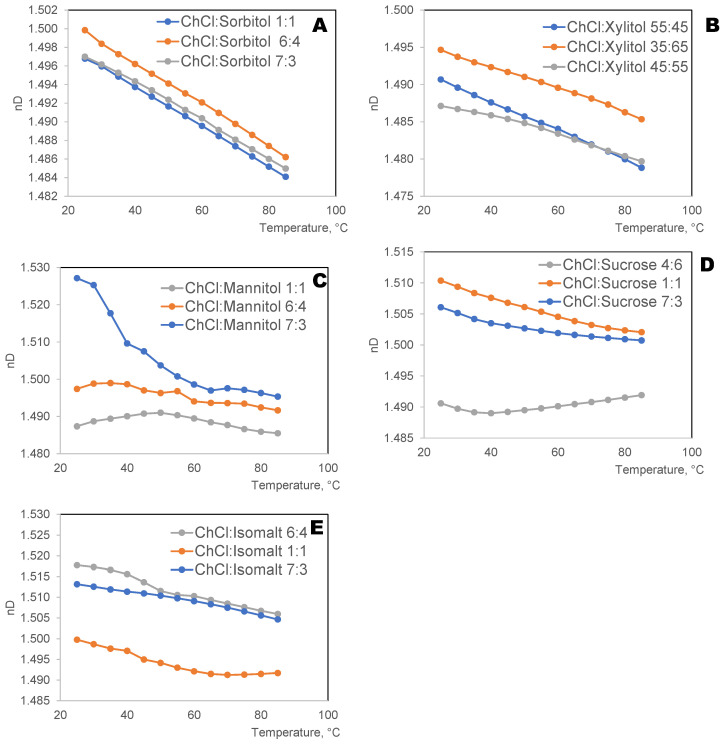
Effect of temperature on the refractive indices of eutectic mixtures of choline chloride (ChCl) with different mole fractions of polyols: sorbitol (**A**), xylitol (**B**), mannitol (**C**), sucrose (**D**), and isomalt (**E**).

The refractive indices of the studied eutectic solvents were measured over the temperature range of 25–85 °C, with a precision of ±1 °C. The results (Figure 5) show that the refractive index of eutectic solvents studied decreases with the increase in the molar ratio of HBAs to HBDs from (1:1 to 4:6). This observation was in line with the results reported by other researchers, for example, by Khajeh et al. [46] and Luan et al. [47].

**Figure 6:**
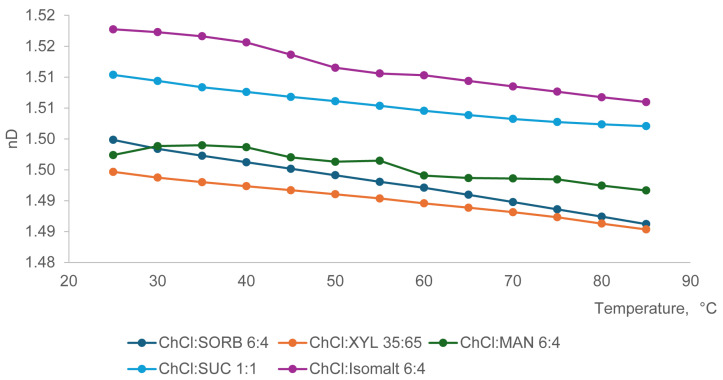
Effect of temperature on the RI of eutectic mixtures of ChCl with various polyols.

The refractive index decreased (Figure 6) in the order from isomalt, followed by sorbitol, sucrose, mannitol, and xylitol, irrespective of the HBA.

#### 2.2.5. Viscosity of Eutectic Solvents

Viscosity is one of the most crucial characteristics of eutectic solvents, but it is also one of the hardest to model. There is currently no standard model in the literature to predict/estimate DES viscosities [48]. At the same time, Hole theory provides a useful explanation for the viscosity of ionic DESs, such as those containing ChCl as HBA [49]. This theory states that ion mobility is limited in highly organised ionic DESs with large hydrogen bonding networks, resulting in increased viscosity.

**Figure 7:**
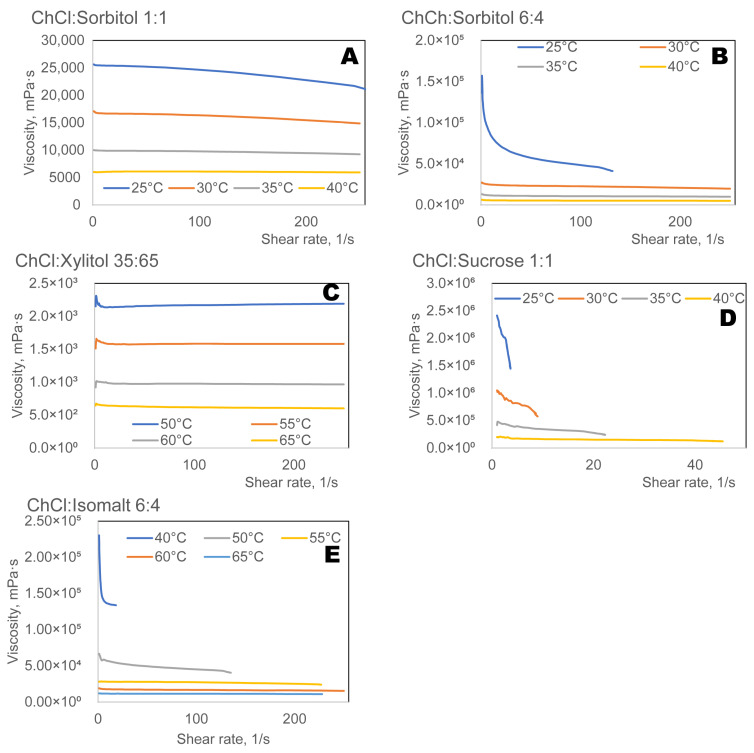
Viscosity of eutectic solvents prepared from choline chloride (ChCl) and various polyols: sorbitol (**A**), xylitol (**B**), mannitol (**C**), sucrose (**D**), and isomalt (**E**).

The viscosity measurements (Figure 7) show relatively high viscosities for all eutectic solvents. The high viscosity of choline chloride–polyol DES comes from strong hydrogen bond networks + ionic interactions, especially when polyols contain many hydroxyl groups [31]. Eutectic solvents with high viscosities can be of interest for topical formulations of drugs [9].

The viscosity of ChCl:ISO was highest, followed by ChCl:SOR, and then ChCl:XYL. These results support reported results in the literature [50,51], that ESs with smaller molecules have lower viscosity. The viscosity of ESs depends on the molar ratio of HBD to HBA [52]. ChCl:sorbitol ESs (Figure 7) show this trend: viscosity increased with the molar fraction of sorbitol.

Sugar-based ESs, including sucrose, are among the most viscous ESs [53]. The viscosity curves of sucrose-based ESs (Figure 7) show that they reached such high viscosities at low temperatures that the rheometer reached its torque limit, so it cannot maintain the imposed shear rate, and rotation stops.

**Figure 8:**
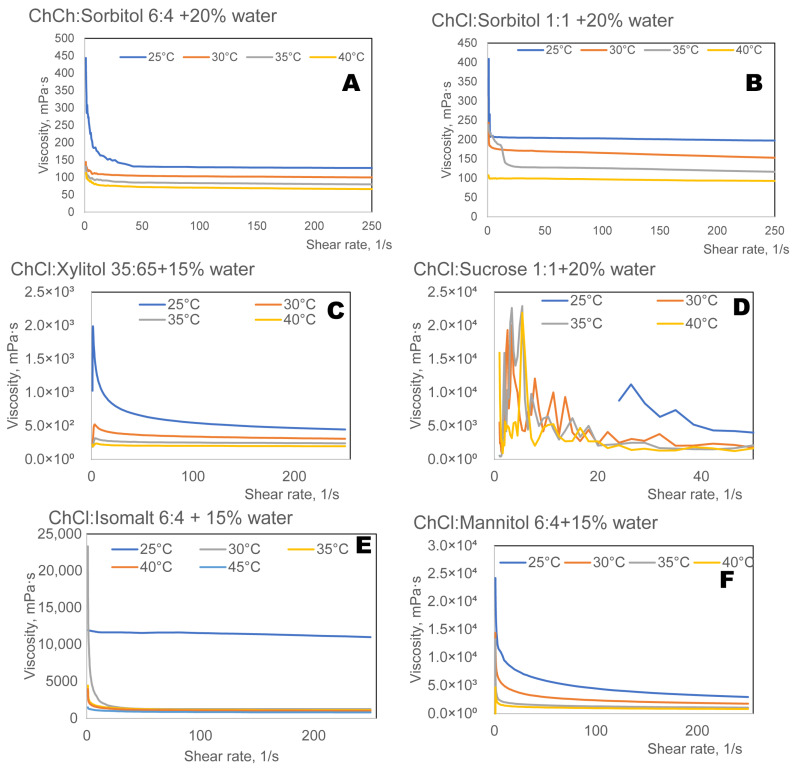
Viscosity of eutectic solvents composed of choline chloride (ChCl) and various polyols with added water (15–20 wt.%): sorbitol (6:4) (**A**) and (1:1) (**B**), xylitol (35:65) (**C**), sucrose (1:1) (**D**), isomalt (6:4) (**E**), and mannitol (6:4) (**F**).

The presence of water in the eutectic system can be an approach to manipulate the viscosity in a non-toxic way [54]. The addition of water to choline chloride and polyol eutectic systems significantly decreased viscosity (Figure 8). The addition of water allows tuning of the viscosity of eutectic solvents for the design of ESs. Using different amounts of water, it is possible to generate viscosities ranging from low-viscosity systems for quick drug release to more viscous formulations for regulated, sustained delivery by varying the composition and molar ratio of the components of ESs.

The choline chloride: sucrose-based ESs showed the behaviour of unhomogeneous semi-solids. Unlike pure liquids, these mixtures often exhibit non-Newtonian behaviour, meaning that their viscosity changes depending on how fast they are being stirred or pushed [55].

Adjusting the viscosity enables optimised drug release profiles from oral dosage forms, thereby improving therapeutic outcomes across a variety of applications.

### 2.3. Influence of Eutectic Solvent on the Apparent Solubility of Poorly Water-Soluble Drugs

Eutectic solvents have emerged as a promising strategy for improving drug solubility, permeability, and overall bioavailability, offering a versatile approach for the formulation of poorly soluble drugs [56,57]. Therefore, the potential of the prepared eutectic solvents to enhance the solubility of ibuprofen, naproxen, and apigenin was investigated. The solubility of these drugs was determined using the widely employed shake-flask method. In this approach, an excess amount of the drug is added to a fixed volume of solvent and continuously stirred at room temperature for a sufficient period to reach equilibrium. After equilibrium is established, the undissolved solid is separated from the solution, and the concentration of the dissolved drug is quantified using an appropriate analytical technique, typically chromatography.

Solubility was determined using the shake-flask method at 40 °C for 24 h, followed by quantification of the dissolved drug using an LC–UV analytical method. Since all the eutectic solvents prepared were semi-solids at room temperature, it was of interest to evaluate their ability to dissolve selected drugs. Depending on the source and experimental conditions, semi-solid systems can still exhibit sufficient solvation capacity due to their unique intermolecular interactions and reduced crystallinity [58]. Therefore, their potential to enhance drug solubility was further investigated.

Depending on the source and experimental setup, ibuprofen (IBU) has a pKa of 4.5–4.9 [9,59] and a water solubility of 59–70 mg/L at 20 °C [60]. Naproxen (NPX) has a pKa of 4.15 [61] and a water solubility of 25 mg/L at 25 °C [62] and 49.6 mg/L at 37 °C [63]. Apigenin (API) has a pKa value between 4 and 5 [64] and a low water solubility of 1.35 mg/L at 25 °C [65].

**Figure 9:**
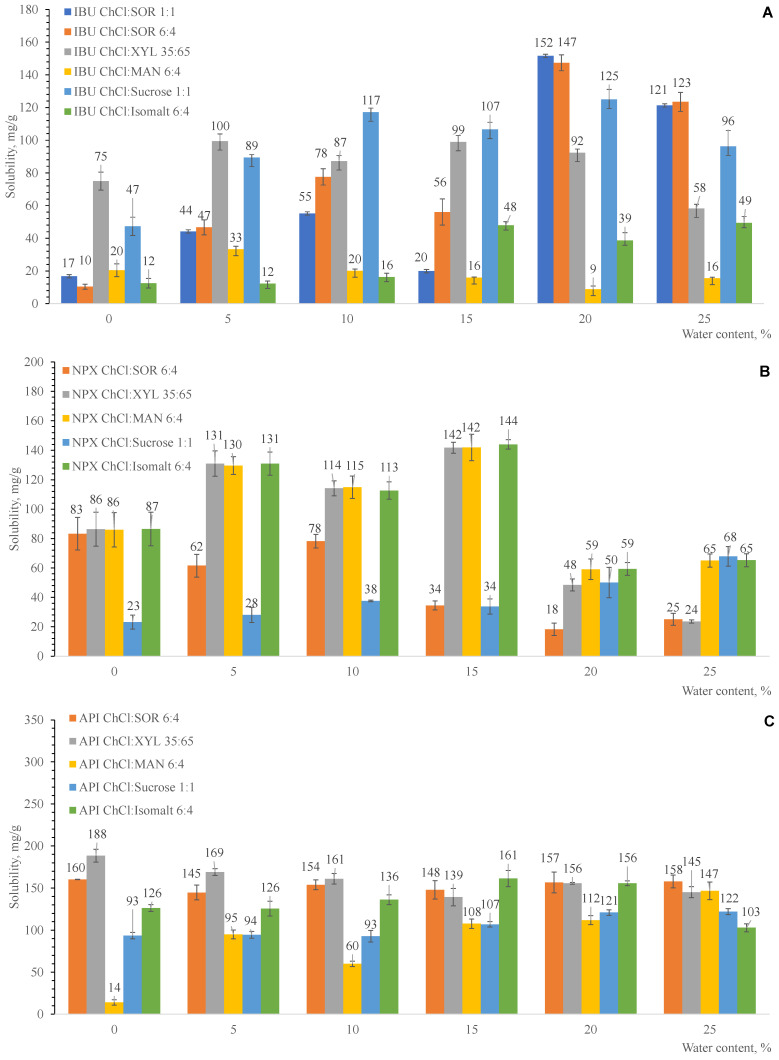
Solubility of IBU (**A**), NPX (**B**), and API (**C**) in eutectic solvents prepared from choline chloride (ChCl) and polyols, and the influence of added water content (expressed in wt.%).

Compared to pure water, the experimental results (Figure 9) clearly show that the eutectic solvent of choline chloride: polyol is a much better solvent for the three model drugs. However, the magnitude of solubility enhancement varies between the compounds: IBU: ~152-fold increase, NPX: ~144-fold increase, and API: ~188-fold increase.

A revision of the literature reveals that the solubility of ibuprofen and naproxen using various eutectic solvents has been investigated previously. For example, Lomba et al. [66] increased the solubility of ibuprofen 21-fold by using a choline chloride:xylitol eutectic solvent. According to the literature, the solubility enhancement of naproxen in neat betaine:propylene glycol ES is more than 3000 times greater than that of water [67].

Additionally, it was observed that in aqueous solutions containing 15–20 wt.% water, the solubility of ibuprofen increased in eutectic solvents containing sorbitol (147–152 mg/g) as the hydrogen bond donor (HBD), while naproxen showed enhanced solubility in systems based on isomalt (144 mg/g), xylitol (142 mg/g), and mannitol (142 mg/g). The highest solubility of apigenin was achieved using xylitol (188 mg/g) as the HBD without the addition of water.

The solubility behaviour of the tested drugs in the prepared eutectic solvents varied depending on their molecular structure. Apigenin, which contains multiple hydroxyl groups, was able to dissolve effectively in the eutectic solvents without the addition of water. This can be attributed to the formation of extensive hydrogen-bonding interactions between apigenin and the components of the eutectic system, such as choline chloride and polyols. In contrast, ibuprofen and naproxen, which possess only a single carboxylic acid group, exhibited limited solubility in the neat eutectic solvents. The addition of small amounts of water improved their solubility by increasing the polarity of the medium, facilitating hydrogen bonding, and helping to disrupt the crystal lattice. These observations highlight the importance of drug polarity and hydrogen-bonding capacity in determining solubility in eutectic solvent systems.

## 3. Materials and Methods

### 3.1. Materials

Ibuprofen (IBU) 70 powder (BASF, Florham Park, NJ, USA); apigenin (API) ≥ 98.0% (HPLC) (Shanghai Macklin Biochemical Technology Co., Ltd., Shanghai, China), and naproxen (NAP) > 99.0% (TCI, Shanghai Development Co., Ltd., Shanghai, China) were used as model drugs in this research.

HBA and HBD compounds: choline chloride (ChCl) > 98.0% (TCI, Tokyo, Japan); sorbitol from Parteck^®^ SI 200 (Merck, Darmstadt, Germany); xylitol 99+% (Thermo Scientific, Acros Organics, Geel, Belgium); mannitol from Parteck^®^ M200 (Merck, Darmstadt, Germany); Isomalt galenIQ™ 800 (Südzucker AG, Obrigheim, Germany); sucrose (Well done, Max. Vilnius, Lithuania).

HYDRANAL™ Methanol dry and Composite 5 reagents were sourced from Honeywell Fluka, Seelze, Germany.

For chromatography analyses, acetonitrile and methanol (HPLC gradient grade purity) were obtained from Fisher Scientific (Loughborough, UK). Formic acid (Suprapur 98–100%) was sourced from Supelco (Darmstadt, Germany). Phosphoric acid (ACS reagent, ≥85 wt % in H_2_O) was obtained from Sigma-Aldrich (Buchs, Switzerland).

### 3.2. Methods

#### 3.2.1. Construction of Solid–Liquid Phase Diagrams

Binary mixtures between polyols (sorbitol (SOR), xylitol (XYL), mannitol (MAN), sucrose (SUC), and isomalt (ISOM)) and choline chloride (ChCl) were prepared in different molar ratios covering the full composition range (at mole fraction intervals of 0.1) by weighing the proper amounts of each pure substance using an analytic balance Radwag (repeatability of 0.01 mg). A total of 90 different capillary tubes containing mole fractions of ChCl and polyols were prepared. A melting point apparatus (SMP30; Cole-Parmer Ltd., Stone, Staffordshire, UK), with a temperature resolution of ±0.1 °C and a ramp rate of 5 °C/min from room temperature up to 250 °C, was used for melting point determination.

#### 3.2.2. Preparation of Eutectic Solvents

In this research, ESs were prepared from choline chloride and different polyols (sorbitol, xylitol, mannitol, sucrose, and isomalt) (Table 3) using thermal mixing and a vacuum oven (OV4-30; Jeiotech Co., Ltd., Daejeon, Republic of Korea). At first, the HBAs and HBDs were mixed with the help of a magnetic stirrer at a temperature detected as the melting point of each ES by the melting apparatus. After one hour, the samples were put in a vacuum oven for 24 h at the same melting temperature.

The molar ratio of the ES components was chosen based on solid–liquid phase diagrams (Figure 2) by additionally taking the point of molar ratio before and after the lowest detected melting point.

#### 3.2.3. Differential Scanning Calorimetry (DSC)

The thermal behaviour of ESs was analysed by DSC (CHIP-DSC 100; Linseis Messgeräte GmbH, Selb, Germany). Ems of 6–11 mg were weighed into crimped aluminium crucibles with a pin-hole lid. The samples were tested at a heating rate of 10 °C/min in the temperature range of 25–200 °C in a nitrogen environment with a nitrogen flow rate of 25 cm^3^/min. Tin and indium were used as reference standards for temperature and enthalpy calibration. For each sample, three duplicate DCS measurements were performed.

#### 3.2.4. Fourier Transform Infrared Spectroscopy (FTIR)

A Nicolet iS20 FTIR (Thermo Fisher Scientific, Madison, WI, USA) was used to obtain the spectra of EMs and their individual pure constituents. A minimum of two measurements from the same surface area were taken with the Attenuated Total Reflectance (ATR) unit. The wave number range was 400–4000 cm^−1^, with a spectrum resolution of 4 cm^−1^. Background absorption was subtracted prior to analysis, and all samples were scanned in triplicate. Spectra were pre-processed by baseline correction, min–max normalisation (0–100), and Savitzky–Golay smoothing (7 points) to reduce noise and intensity variations.

#### 3.2.5. Karl Fischer (KF) Titration

Water content was determined by volumetric Karl Fischer titration. The accurately weighed samples of approximately 250 mg were analysed using a V10S KF titrator (Mettler Toledo AG, Bern, Switzerland) with HYDRANAL™ Methanol dry and Composite 5 reagents (Honeywell Fluka, Seelze, Germany). Triplicate measurements were performed, and the water content (wt.%) was presented as averages ± S.D. (*n* = 3).

#### 3.2.6. pH Value Measurement

The pH values for each DES were determined with a pH/conductivity meter (SD23-KIT; Mettler Toledo GmbH, Greifensee, Switzerland) within the pH measuring range of 0–9 at room temperature. The instrument was calibrated using standard pH buffer solutions. All measurements were carried out in triplicate, and the results were expressed as average values ± standard deviation.

#### 3.2.7. Loss on Drying (LoD)

The moisture content of DES was measured by thermogravimetric analysis (LoD). Approximately 1–1.5 g of samples were exposed to a constant temperature of 230 °C, and the weight loss was recorded using a Halogen Moisture Analyzer (HX204; Mettler Toledo AG, Greifensee, Switzerland). Measurements were performed in triplicate, and LoD results (wt.%) were reported as average values ± standard deviations (S.D.) (*n* = 3).

#### 3.2.8. Refractive Index

About two to three droplets of DES were placed on the measuring prism and sealed for each measurement. The Abbemat 350 Performance Plus refractometer (Anton Paar OptoTec GmbH, Seelze-Letter, Germany) was stabilised for approximately one minute in order to reach the desired temperature of 25 °C (±0.01 °C). Following every measurement, the prism was cleaned three times using ultrapure water before wiping with lens paper. At least three measurements were averaged to provide the reported values.

#### 3.2.9. Viscosity Measurements

The viscosity curves of the samples were measured using a Modular Compact Rheometer (MCR 102e; AntonPaar, Graz, Austria) at temperatures of 25, 30, 35 and 40 °C, covering a shear rate range of 1–250 s^−1^. Prior to each measurement, the samples were equilibrated at the target temperature for 10 min. A fresh sample was loaded for each measurement to ensure that all the samples had the same thermal and shear history. All rheological measurements were performed using a parallel-plate geometry (PP50) with a screw-on stage of 50 mm diameter and a fixed plate gap of 1 mm.

#### 3.2.10. Solubility Measurement (Shake-Flask Method)

The solubility studies of ibuprofen, naproxen and apigenin were performed according to the methodology described by Nica et al. [57] using the shake-flask method. Briefly, to reach equilibrium solubility, an excess amount of drug was added to ≈500 mg of each ES in a 1.5 mL Eppendorf tube; the samples were vortexed and kept for 24 h at 40 °C in a water bath with magnetic stirring. The samples were further centrifuged at 20,000 rpm for 15 min to separate the undissolved drug, after which ≈50 mg of supernatant was transferred to a 5 mL volumetric flask and diluted to volume with acetonitrile. Prior to quantification, the samples were filtered using 0.45 µm PVDF syringe filters.

#### 3.2.11. Drug Quantification

The samples were further analysed with HPLC (Vanquish LC-UV system; Thermo Scientific, Dionex Softron GmbH, Germering, Germany) equipped with a quaternary pump, a thermostated autosampler, a column oven, and a Diode Array Detector (DAD). All samples were analysed by using a C18 column (Zorbax Eclipse Plus C18; 2.1 × 150 mm, 5 µm; Agilent, Santa Clara, CA, USA).

For ibuprofen quantification, the mobile phase was isocratic and consisted of 55% acetonitrile and 45% of a formic acid aqueous solution (2%). The analysis was performed at a flow rate of 1 mL/min at a temperature of 45 °C.

For naproxen quantification, the mobile phase was isocratic and consisted of 50% acetonitrile and 50% of a 0.4% phosphoric acid solution. The analysis was performed at a flow rate of 0.5 mL/min and a chamber temperature of 35 °C.

For apigenin quantification, the mobile phase was isocratic and consisted of 55% acetonitrile and 45% formic acid solution (0.1%).

The detection wavelengths were set at 222 nm for ibuprofen and naproxen, and 269 nm for apigenin. All solutions were injected in triplicate (10 µL). The quantification of all model compounds dissolved in each ES system was performed by interpolating peak areas against calibration curves, which were constructed using standard solutions within the concentration range of 0.1–100 µg/mL for ibuprofen (C = (Area − 5.3483)/344.63; R^2^ = 0.999)), 5.0–200 µg/mL for naproxen (C = (Area − 4.0459)/4065.5; R^2^ = 0.999)), and 1–112 µg/mL for apigenin (C = (Area − 0.0278)/445.95; R^2^ = 0.999)). The results were expressed as mg of model compound per gram of eutectic solvent.

## 4. Conclusions

This study demonstrates that choline chloride–polyol-based eutectic solvents can be successfully prepared via simple thermal mixing, and their physicochemical properties can be effectively characterised using FTIR, DSC, pH measurements, refractive index, and moisture content analysis.

Furthermore, these pharmaceutically acceptable eutectic systems were shown to significantly enhance the solubility of ibuprofen, naproxen, and apigenin, addressing a key challenge in improving their bioavailability and therapeutic efficacy. Collectively, these findings highlight the potential of choline chloride–polyol eutectic solvents as versatile and effective media for the formulation of poorly soluble drugs.

The results demonstrate that the solubility of ibuprofen, naproxen, and apigenin is dependent on the type of polyol and its mass fraction in the eutectic system. Additionally, the significant role of water as a modifier in polyol-based choline chloride eutectic systems was demonstrated, where regulated water addition can alter the viscosity and drug solubility of the system. In comparison to anhydrous systems, ibuprofen and naproxen were more than 100 times more soluble at 15–20 wt.% water concentrations; viscosity dropped with increasing water content. Because of their tunability, eutectic solvents can be used in a wide range of drug delivery applications, from formulations with controlled drug release over extended periods.

The solubility of drugs in eutectic solvents based on choline chloride and polyols depends strongly on their molecular structure and hydrogen-bonding capacity. Highly hydroxylated compounds like apigenin can dissolve directly in the eutectic solvents, whereas less polar drugs such as ibuprofen and naproxen require the addition of water to enhance solubility. This demonstrates that the effectiveness of eutectic solvents as solubilizing agents is influenced by both drug polarity and the potential for hydrogen-bonding interactions.

## Figures and Tables

**Table 1:** Eutectic points of ChCl: polyol mixtures.

ES	Eutectic Point	
	Experimentally Detected	Literature Values
ChCl:SOR	6:4 (54.9 °C)	1:1 (46.3 °C) [30] 1:1 (25 °C) [31]
ChCl:XYL	4:6 (54.7 °C)	55:45 (33.4 °C) [30] 1:1 (25 °C) [31] 33:66 to 66:33 (45 °C) [32]
ChCl:MAN	6:4 (82.3 °C)	5:5 (137 °C) [30]
ChCl:SUC	1:1 (97.3 °C)	1:1 (80 °C) [33]
ChCl:ISOM	6:4 (64.6 °C)	Not found

**Table 2:** Loss on drying, water content by Karl Fischer, and pH values of eutectic solvents.

DES	LOD ± STD, %	Moisture (KF) ± STD, %	pH ± STD
ChCl:SOR 1:1	2.5 ± 0.4	0.85 ± 0.02	7.3 ± 0.1
ChCl:SOR 6:4	2.1 ± 0.2	0.74 ± 0.02	7.6 ± 0.5
ChCl:Xyl 35:65	2.4 ± 0.5	0.57 ± 0.06	7.7 ± 0.2
ChCl:Man 6:4	4.6 ± 0.7	1.37 ± 0.10	7.6 ± 0.3
ChCl:Suc 1:1	2.4 ± 0.5	1.51 ± 0.38	7.5 ± 0.4
ChCl:Iso 6:4	3.3 ± 0.2	2.66 ± 0.10	6.7 ± 0.2

**Table 3:** Composition of ESs.

ES	HBA	HBD	Molar Ratio
ChCl:SOR	Choline chloride	Sorbitol	1:1; 6:4; 7:3
ChCl:XYL		Xylitol	4:6; 1:1; 6:4
ChCl:MAN		Mannitol	6:4; 1:1; 7:3
ChCl:SUC		Sucrose	4:6; 1:1; 3:7
ChCl:ISOM		Isomalt	1:1; 6:4; 7:3

## Data Availability

The original contributions presented in this study are included in the article/Supplementary Materials. Further inquiries can be directed to the corresponding author.

## Supplementary Materials

The following supporting information can be downloaded at: https://www.mdpi.com/article/10.3390/ijms27073110/s1.

## Abbreviations

The following abbreviations are used in this manuscript:

ES	Eutectic solvent
DES	Deep eutectic solvent
ChCl	Choline chloride
SOR	Sorbitol
XYL	Xylitol
MAN	Mannitol
SUC	Sucrose
ISO	Isomalt
IBU	Ibuprofen
NPX	Naproxen
API	Apigenin
DSC	Differential Scanning Calorimetry
FTIR	Fourier Transform Infrared Spectroscopy
LOD	Loss on Drying
KF	Karl Fischer
