# Supplementary material for: A Green Innovative Approach for Solubility Enhancement of Poorly Water-Soluble Drugs Using Choline Chloride–Polyol Eutectic Solvents
Source: Int J Mol Sci. 2026 Mar 29;27(7):3110. doi: 10.3390/ijms27073110 (PMC13072729; doi:10.3390/ijms27073110)
Supplement: Supplementary file 1 [file ijms-27-03110-s001.zip › ijms-4224655-supplementary.pdf]

Supplementary

A green innovative approach for solubility enhancement of poorly water-soluble drugs using choline chloride–polyol eutectic solvents

Liga Petersone *, Rihards Mahinovs, Zoltán Márk Horváth and Valentyn Mohylyuk

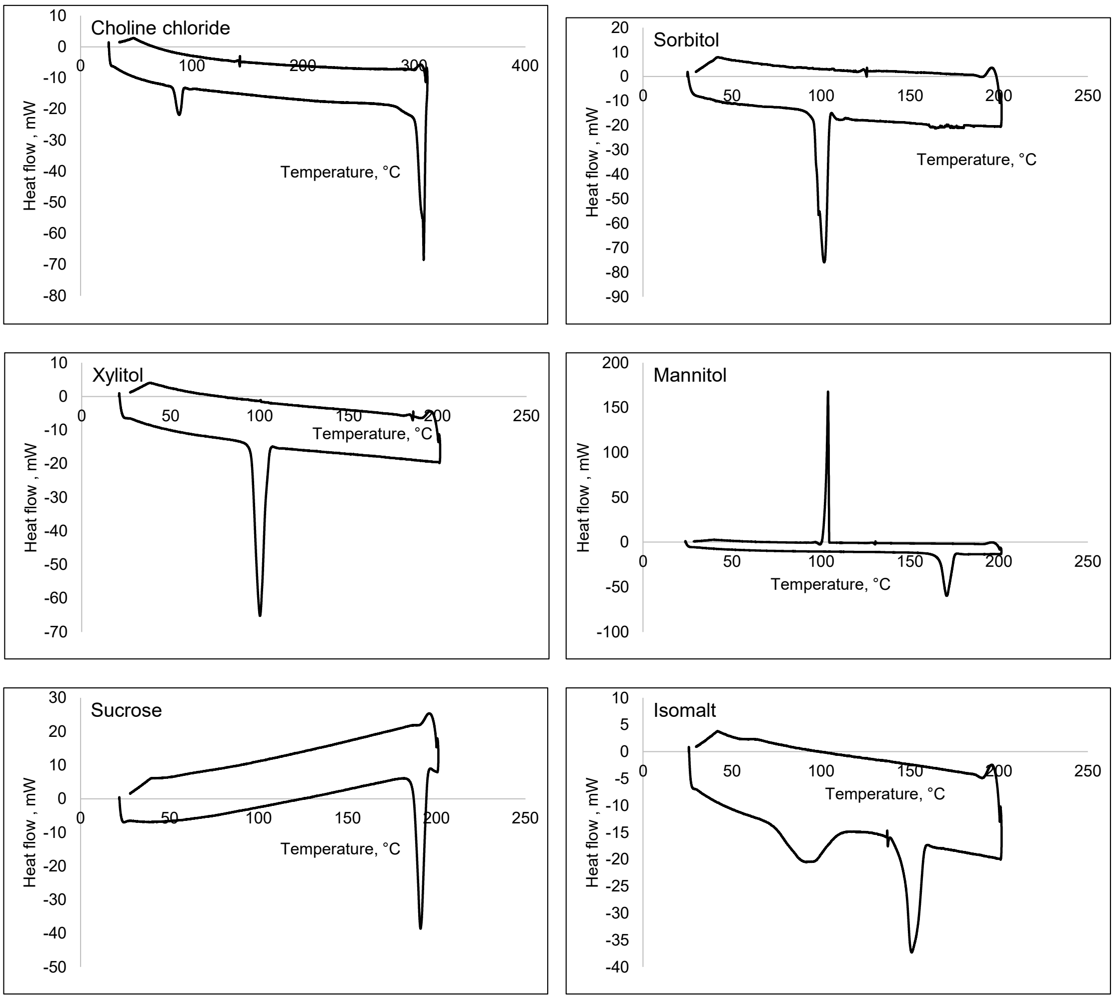

Figure S1. DSC curves of choline chloride and pure polyols.

Table S1. Binary mixture mole fraction compositions studied in this work.

	**Mole fraction**	**Melting Temperature, °C**				
		**Sorbitol**	**Xylitol**	**Mannitol**	**Sucrose**	**Isomalt**
**Choline chloride**	0.00			301.2		
	0.10	214.4	132.4	178.5	179.5	101.3
	0.40	54.9	56.4	82.3	99.7	64.6
	0.50	68.5	58.8	85.5	97.3	66.8
	0.55	62.6	54.7	83.4	102.8	67.9
	0.60	72.8	62.3	82.3	105.2	74.2
	0.70	75.4	65.7	83.2	117.5	80.6
	0.80	86.3	87.6	127.5	135.8	102.3
	1.00	97.0	95	169.5	191	138

Table S2. Visual characterization of eutectic solvents prepared.

Eutectic solvent	Molar ratio / Picture		
ChCl:SOR	1:1<br>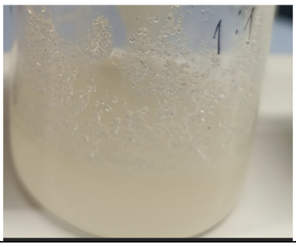	6:4<br>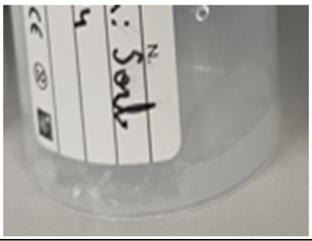	7:3<br>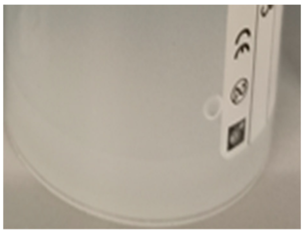
ChCl:XYL	35:65<br>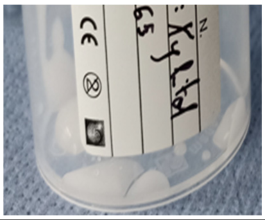	45:55<br>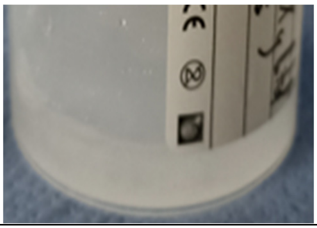	55:45<br>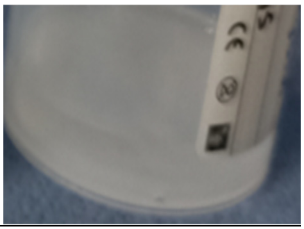
ChCl:MAN	1:1<br>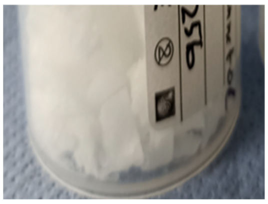	6:4<br>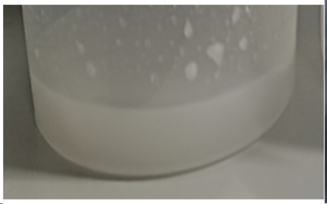	7:3<br>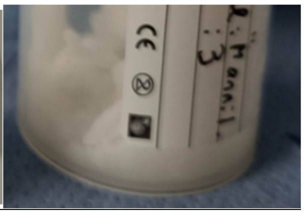
ChCl:SUC	4:6<br>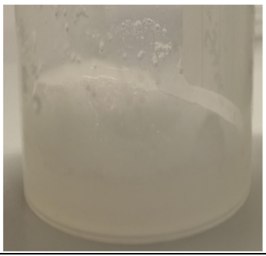	1:1<br>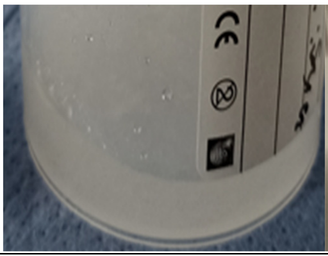	6:4<br>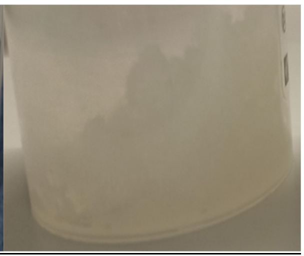
ChCl:ISO	1:1<br>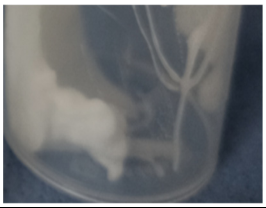	6:4<br>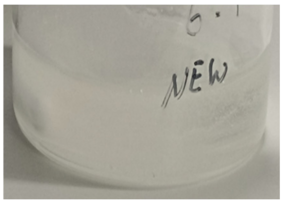	7:3<br>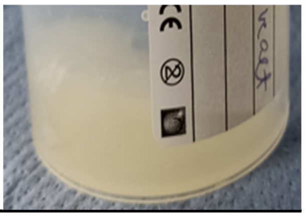
